# Exosomes cloak the virion to transmit Enterovirus 71 non-lytically

**DOI:** 10.1080/21505594.2019.1705022

**Published:** 2019-12-28

**Authors:** Jiaqi Gu, Jing Wu, Daihua Fang, Yang Qiu, Xinran Zou, Xiaonan Jia, Yiqian Yin, Li Shen, Lingxiang Mao

**Affiliations:** a Department of Laboratory Medicine, The Affiliated People’s Hospital, Jiangsu University, Zhenjiang, China; b Department of Immunology, Jiangsu Key Laboratory of Laboratory Medicine, School of Medicine, Jiangsu University, Zhenjiang, China; c Clinical Laboratory, Xuzhou children’s hospital, Xuzhou, China; d State Key Laboratory of Virology, Wuhan Institute of Virology, Center for Biosafety Mega-Science, Chinese Academy of Sciences, Wuhan, China; e Clinical Laboratory, Zhenjiang Center for Disease Control and Prevention, Jiangsu, China

**Keywords:** EV71, non-lytically, exosomes, Density, EXO-EV71

## Abstract

Enterovirus 71 (EV71) is a non-enveloped virus and it can be released from host cells through a traditional cytolytic manner. Now, we showed EV71 could be spread non-lytically between cells during early viral infection. In order to explain this phenomenon, we separated supernatant fluids of rhabdomyosarcoma (RD) cells cultures infected with EV71 by isopycnic gradient centrifugation. Two populations of virus particles were morphology indistinguishable by transmission electron microscope (TEM). It showed that some EV71 particles were wrapped inside extracellular vesicles which were verified to be exosomes by immunoassay and morphologic analysis. In addition, exosomes containing viral RNA were shed in plasma of EV71-infected encephalitis in children. Our findings indicate that the “non-enveloped” EV71 virions could be wrapped within exosomes which promote their spread in the absence of cell lysis.

**Abbreviation**: EV71: enterovirus 71; EXO: exosome; RD: rhabdomyosarcoma; TEM: transmission electron microscope; HFMD: hand, foot, and mouth disease; HIV: immunodeficiency virus; HCV: hepatitis C virus; HTLV: Human T-cell lymphotropic virus; HAV: hepatitis A virus; MOI: multiplicity of infection; EVs: extracellular vesicles; VP1: viral capsid protein 1; NTA: nanoparticle tracking analysis; CNS: central nervous system

## Introduction

EV71 is a positive single-stranded RNA virus that belongs to the family *Picornaviridae*, genus *Enterovirus*, species Enterovirus A. The EV71 particle is symmetrical with a 20–30 nm icosahedral capsid. EV71 is considered as a classic non-enveloped virus which had to be spread after lysis of host cells. It can cause hand, foot, and mouth disease (HFMD) which is usually self-limiting but highly contagious in children under 5 years old [1]. In addition to HFMD, EV71 has been shown to have the ability to infect the central nervous system (CNS) and can cause various neurological complications, including aseptic meningitis, brainstem encephalitis, acute flaccid paralysis, neurogenic pulmonary edema, delayed neurodevelopment, and reduced cognitive function [2]. It has been proposed that there are two possible routes by which EV71 reaches the CNS: the virus either enters the CNS from the blood across the blood-brain barrier (BBB) or is transmitted to the CNS through peripheral nerves via retrograde axonal transport [3]. As for the first route, concerning the vehicle for the virus, although the viral RNA could be detected in leukocytes, the majority of the infectious virions in the bloodstream were present in the plasma, indicating that infecting leukocytes was probably not the vehicles for transportation into the CNS. The EV71 carrier from the bloodstream towards the nervous system is still largely unknown.

Exosomes (30-150nm) are one subtype of extracellular vesicles (EVs) secreted into extracellular space by most cells. The exosomes can transmit their contents, such as protein, lipid, and RNAs, to recipient cells or distal tissues and participate in various biological processes [4]. In addition to performing many biological functions [5], exosomes could transfer viral components including viral RNA, proteins and other host functional genetic elements between cells. The role of exosomes in viral infection usually depends upon the cells of origin. Besides, several non-envelop viruses, poliovirus, hepatitis A and coxsackievirus B3 have also been identified in EVs being released from intact cells [6]. However, to date, whether the EV71 virions were wrapped in the exosomes are undetermined. The exosomeal content and the exosomal contributions to the pathogenesis of EV71 remain largely unexplored.

In this study, we demonstrate that EV71 virions could be internally enclosed in the exosomes (EXO-EV71) *in vitro* which may play an important role in viral dissemination. Moreover, we show that EV71 RNA could be detected in the exosomes (EXO-EV71-RNA) which were isolated from the plasma of EV71-infected encephalitis in children. These findings are the first to elucidate the intact EV71 virions were transported in the exosomes, provide new insights to the pathogenic mechanisms of EV71.

## Materials and methods

### Cells and viruses

RD cells (human rhabdomyosarcoma cells) were cultured in DMEM medium supplemented with 10% fetal bovine serum (FBS). The cell lines were obtained from ATCC. Cells were infected with EV71 at multiplicity of infection (MOI) of 1 and incubated for 1 h at 37°C/5%CO_2_. Then, cells were rinsed and further incubated with pre-warmed DMEM supplemented with 5% FBS which had been ultracentrifuged to remove exosomes. The cells were collected and cracked by three freeze-thawing cycles and centrifuged at 4000× rpm at 4°C for 10 min to remove cellular debris. Infectious virus titers were determined by TCID_50_ assay as described elsewhere [7].

### Human samples and clinical information

The collection of all the samples and clinical information were approved by the Ethics Committee of Jiangsu University and Xuzhou Children’s Hospital and written informed consent was obtained from parents or legal guardians of all children.

### qRT-PCR assay for EV71 RNA

Total RNA were extracted by using Trizol reagent (Invitrogen). EV71 RNA was determined by a SYBR green One-step qRT-PCR assay (Bio-Rad). The primer sequences of EV71 were performed as described previously [8].

### Immunofluorescence assay

RD cells were seeded at 10,000 cells per well in optical-bottom 96-well plates coated in 200μL 10%FBS DMEM at 37°C overnight, incubated with EV71 for 1 h and rinsed with PBS three times, then coated in 200 μL 2%FBS DMEM. 10 μL of 10μM SYTOX^TM^ Blue Nucleic Acid Stain (Life Technologies) was added to the well to incubate for 30 min without rinse before taking images by Cytation^TM^5 (Bio Tek).

### Isopycnic gradient centrifugation

Cell-culture supernatant fluids were centrifuged at 1,000 × g at 4°C for 10 min to remove cells and debris, and then centrifuged at 10,000 × g at 4°C for 30 min twice to remove subcellular fraction and collected the EVs after at 100,000 × g for 1 h at 4°C by ultracentrifugation. The EVs was resuspended in PBS, loaded onto an 8-40% iodixanol (Opti-prep) step gradient and centrifuged at 150,000 × g in an SW32TI Beckman Coulter rotor for 24 h at 4°C. One-milliliter fractions were collected, and density was determined with a refractometer.

### Transmission electron microscopy (TEM)

2.5 μL Samples were adsorbed on the surface of a glow-discharged 400 mesh carbon-coated copper grid for 5 min and make the grid fixed in 1% glutaraldehyde with 0.15M phosphate buffer (pH7.4) for 1 min, afterward rinsing with deionized water and staining with 3% ammonium molybdate pH 7.0.

### Immunoblotting

Cells were lysed with radioimmunoprecipitation assay (RIPA) buffer (Kangwei Century). Immunoblots were performed with standard procedures and the indicated antibodies. Protein bands were detected by Image Quant LAS 4000 mini (GE Healthcare).

### Exosomes isolation from human samples

Stools were resuspended by normal saline and centrifuged at 1,000 × g at 4°C for 10 min to remove residue. Exosomes from plasma and pretreated stools were concentrated by Total Exosome isolation Regent (from plasma, Invitrogen^TM^), and then incubated with antibody-coated Dynabeads (CD9.CD81.CD63) (Exosome Isolation Kit Pan, MACS) 24 hr at 4°C on a test tube rolling machine. Collecting the beads after washing three times with PBS containing 1mg/mL BSA and resuspending beads in PBS.

### Proteinase K treatment

Exosomes were incubated with 20 μg/mL proteinase K (Invitrogen) at 37°C for 30 min and the proteinase K activity was inhibited by adding 0.1 μM PMSF and SDS loading buffer for 5 min at 98°C. Addition of 0.1% Triton X-100 disrupted the lipid membrane resulted in complete digestion of all protein [9].

### Statistical analysis

Data were analyzed by GraphPad Prism software (version 6.01). P value<0.05 was considered significant.

## Results

### *EV71 particles are released non-lytically* in vitro

**Figure 1.**
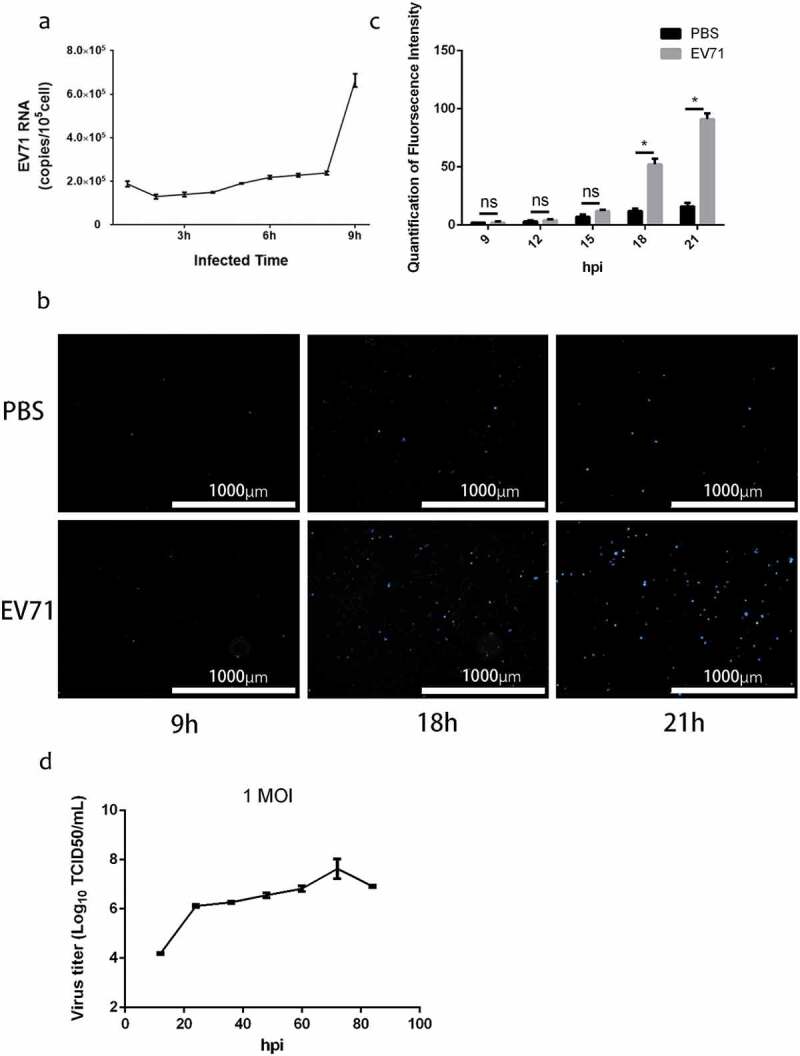
Enterovirus 71 Exit Cells Non-lytically In Vitro. (a) viral RNA was detected in culture supernatant after infected 1–9 h by qRT-PCR assay (mean±SD; three independent experiments). (b) infected cells incubated with SYTOX^TM^ Blue Nucleic Acid Stain to monitor plasma membrane intactness. c, quantification of fluorescence intensity in Figure 1(b) (mean±SD; three independent experiments; *p ≤ 0.05). Statistical analysis were performed using unpaired two-tailed student’s t-test. D, single-step growth curve of RD cells infected with EV71 at MOI of 1. Supernatants were collected from infected cells at the indicated time points, and virus titers were calculated by standard TCID_50_ assay.

To investigate the viral life cycle in RD cells, we detected EV71 RNA in culture supernatant by qRT-PCR after infection at MOI of 1 for 1 h, meanwhile, monitored the integrity of infected cells continuously by the fluorescence of SYTOX Blue Stain, a cell-impermeable dye that binds to nucleic acid with high affinity when the cell membrane is damaged. Remarkably, the results indicated EV71 RNA resolved from culture supernatant with intact membrane could be detected at 9 hpi (Figure 1(a)) while the integrity of the infected cells did not been damaged significantly until 18 hpi (Figure 1(b,c)). The growth curve of EV71 at MOI of 1 also indicated viral titer had obviously increased from 12 to 24 hr. The results proposed that a fraction of the EV71 could be egressed to extracellular space without cell lysis.

### *EV71 virions could be wrapped within exosomes* in vitro

**Figure 2.**
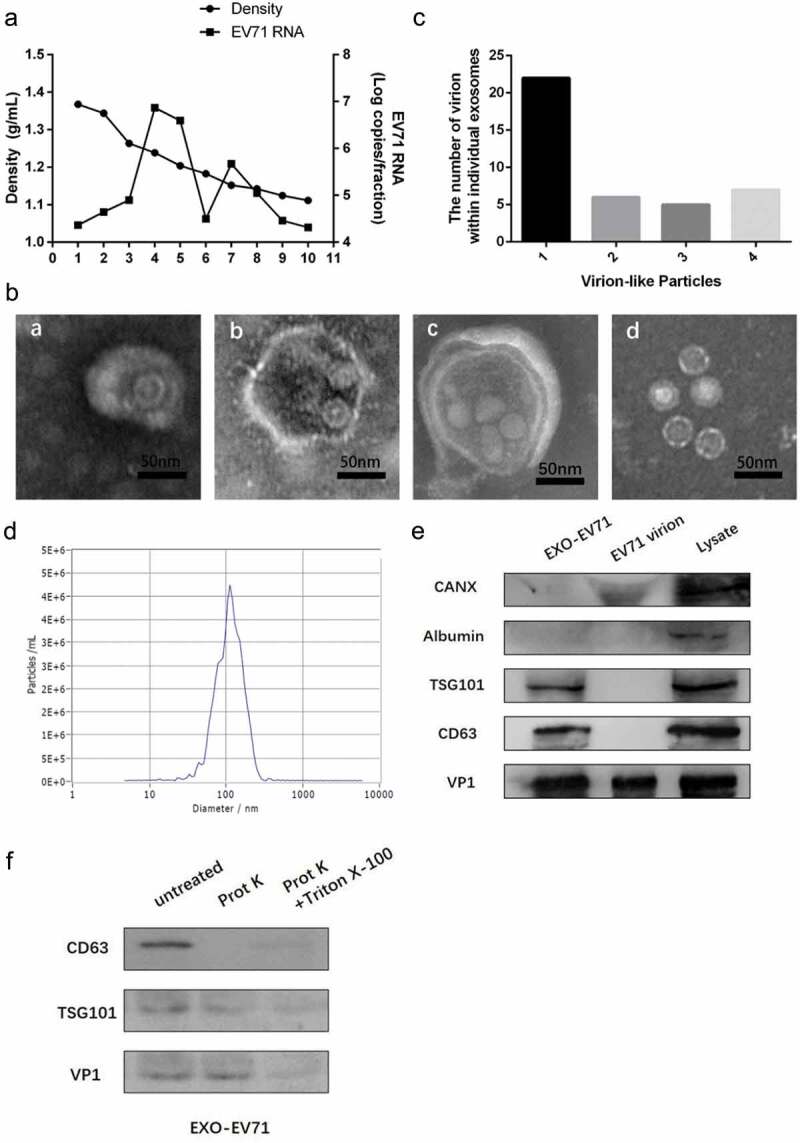
EV71 virions could be wrapped within exosomes *in* *vitro*. (a) buoyant density of EV71 particles released by RD cells in iodixanol gradients. Exosome-like vesicles wrapping EV71 particles (EXO-EV71) gathered in 1.10–1.12g/mL (fraction 7–8), non-enveloped EV71 particles gathered in 1.18–1.26g/mL (fraction 4–5). (b) TEM images of EXO-EV71 (a-c, fraction 7–8 in A) and non-enveloped EV71 (d, fraction 4–5 in A). c, distribution of the number of virion-like particles contained within individual exosomes. d, distribution of EXO-EV71 size measured by nanoparticle NTA. e, immunoblots of EV71 capsid proteins (VP1) and exosomes positive marker (CD63 and TSG101) and negative marker (CANX and Albumin) in lysate of EV71-infected cells, EXO-EV71 and EV71 particles. f, immunoblot analysis of CD63, TSG101 and VP1 in EXO-EV71 exposed to proteinase K with or without detergent (0.1% Triton X-100, used to ensure degradation of both surface and internal components).

To explore how EV71 spreads from intact cells, we separated EV71 from culture supernatant of EV71-infected RD cells by isopycnic gradient centrifugation. Culture of RD cells infected with EV71 contained two populations harboring high expression of EV71 RNA (Figure 2(a)). One population was at a low density (1.10–1.12 g cm^−3^, fraction 7–8) consistent with small extracellular vesicles, whereas the other population (fraction 4–5) was at the density expected for EV71 (1.18–1.26 g cm^−3^).

Notably, two populations showed different morphologies. The population at high density (1.18–1.26 g cm^−3^) contained plentiful 27–30 nm EV71 particles (d in Figure 2(b)). The population at a low density (1.10–1.12 g cm^−3^) contained some 30–150 nm vesicles (a-c in Figure 2(b)) wrapping between one and four virus-like particles with morphology similar to 27–30 nm EV71 particles in dense fractions (d in Figure 2(b)), and besides empty exosomes, the distribution of the number of virion-like particles inside one vesicle is shown in Figure 2(c).

Furthermore, Nanoparticle Tracking Analysis (NTA) revealed these vesicles with low density (1.10–1.12 g cm-3), with diameter concentrated on the size of 101.8 nm (Figure 2(d)), which was consistent with exosomes in dimension. Besides, exosomes markers CD63 and TSG101 and viral protein VP1 could be detected in these vesicles by immunoblots, while albumin and calnexin(CANX) as a negative control to confirm no cell debris in exosomes preparation (Figure 2(e)). These results suggested that EV71 virions were wrapped within exosomes in the supernatant fluids of EV71-infected RD cells.

To confirm whether EV71 particles were actually enclosed in the exosomes, but not on the exosomal surface, proteinase K was used to nonspecifically digest proteins and Triton X-100 was used to destroy lipid membrane. The result indicated that CD63 on the exosomal membrane was digested by proteinase K but the membrane-binding protein TSG101 and viral protein VP1 were protected from proteinase K in the exosomes. However, addition of 0.1% Triton X-100 which disrupted the lipid membrane of the exosomes resulted in completed digestion of all protein (Figure 2(f)). These further evidences indicated EV71 could be released within the exosomes which verified by the size, morphology, and markers *in vitro*.

### Exosomes containing viral RNA (EXO-EV71-RNA) existed in patients’ plasma

**Figure 3.**
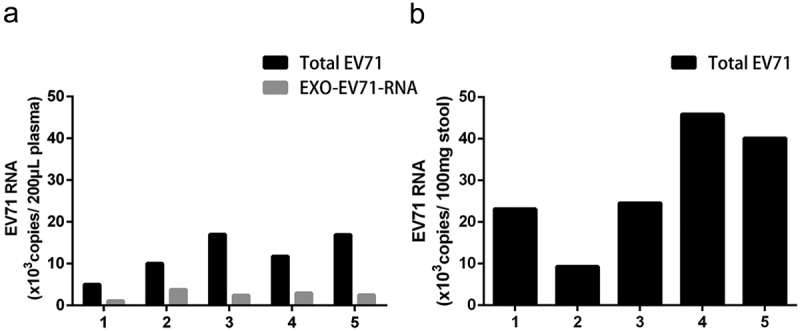
Exosomes Containing Viral RNA (EXO-EV71-RNA) Existed in Patients’ Plasma. (a) viral RNA could be detected in patients’ plasma and in the exosomes of the plasma. (b) viral RNA was positive in patients’ stools.

To investigate whether EV71 might be carried in exosomes *in vivo*, the plasma and stool samples from five EV71 infected severe HFMD patients with viral encephalitis were collected and isolated exosomes. The exosomes were isolated from patients’ plasma but not from the stool because the exosomes may be digested by bile while through the biliary tract to the gut [10]. Then, we extracted the viral RNA directly from clinical samples and from the exosomes of patients’ plasma, respectively. The qRT-PCR results indicated EV71 RNA existed in the plasma and stool samples of these severe HFMD patients (Figure 3(a,b)). Importantly, EV71 RNA was also in the exosomes (EXO-EV71-RNA) circulating in the plasma (Figure 3(a)).

## Discussion

Since EV71 involves the central nervous system (CNS) and can cause serious and potentially fatal neurological complications, it has emerged as a major concern among pediatric infectious diseases [11]. However, the mechanisms involved in its neuropathogenesis are still largely unknown.

Previously, the non-enveloped members of *Picornaviridae* have been thought to be released from the infected cells exclusively via cytolysis. Recently, poliovirus was shown that it could be spread by the autophagy pathway between cells in the absence of lysis [12]. Coxsackievirus B can be encapsulated in cells’ membranes to escape the cells in mitophagosomes [13]. HAV could be cloaked in host-derived membranes and spread non-lytically. The enveloped HAV was less susceptible to antibody-mediated neutralization than naked HAV [10].

We and others have already proposed that exosomes from EV71-infected cells containing viral components have infectivity [8,14]. Our previous study demonstrated exosomes released from EV71-infected cells which could deliver viral RNA and structural protein VP1 are partially resisted to antibody neutralization [8]. However, Fu et al reported that the exosomes could only deliver viral RNA without viral protein and selectively package miR-146a which facilitated viral infection by suppressing type I interferon responses [14]. In fact, it is not clear whether the exosomes contain intact virions yet. Compared with these previous studies, now we used a better method: isopycnic gradient centrifugation with high specificity to separate exosomes from supernatant according to the guidelines [15]. We found intact EV71 virions could be wrapped within exosomes which were verified by TEM, NTA, immunoblotting, and digestion of proteinase K.

Upon infection, viral RNA is recognized as a pathogen-associated molecular pattern (PAMPs) by Toll-like receptors (TLRs) to induce host immunity and inflammation. During EV71 infection, the hepatocyte growth factor-regulated tyrosine kinase substrate (HRS) facilitates the TLR7 complex assembly, leading to the induction of inflammatory and immune responses [16]. An in-depth analysis of the plasmablast-derived IgG mAbs from EV71-infected children unraveled the epitopes for the neutralizing antibodies induced by natural infection [17]. Wu et al proposed that the full particle (FP) VP1 subunit in the EV71 vaccines was the major immunogen conferring wide-range neutralizing efficacy. However, it has implied that the exosomes derived from EV71-infected cell are partially resisted to antibody neutralization [8]. Considering EXO-EV71, further investigations into the prophylactic and therapeutic methods targeting the infectious exosomes should be done in the future.

In addition, our findings showed exosomes containing EV71 RNA (EXO-EV71-RNA) circulated in encephalitis patients’ plasma, although EV71 could migrate to the brainstem via retrograde axonal transport [3] and prohibitin (PHB) was involved in the entry of EV71 virus into motoneuron cells, where EV71 could enter the CNS [4]. There may be multiple routes used for EV71 to enter the nervous system, because EV71 that shed into the bloodstream also might invade the CNS across the BBB. There are increasing studies suggested EVs including exosomes could cross the BBB [18–20]. Based on this, we hypothesize that the exosomal pathway maybe have a partial role in neuropathogenesis of EV71 infection. Further investigations are necessary to determine the relevance between EXO-EV71-RNA and CNS infection.

In conclusion, our results show a previously unknown mechanism of EV71 egress from infected cells. These findings that EXO-EV71 was secreted from infected cells and EXO-EV71-RNA was in patients’ plasma provide new insight into further studies of the function of EXO-EV71 in EV71-infected neurological complications. It is an enlightenment for us to consider the function and mechanism(s) of exosomes in viral CNS infection, though the detail conjectures require further study for confirmation.
